# Perdeuteration, crystallization, data collection and comparison of five neutron diffraction data sets of complexes of human galectin-3C

**DOI:** 10.1107/S2059798316015540

**Published:** 2016-10-28

**Authors:** Francesco Manzoni, Kadhirvel Saraboji, Janina Sprenger, Rohit Kumar, Ann-Louise Noresson, Ulf J. Nilsson, Hakon Leffler, S. Zoë Fisher, Tobias E. Schrader, Andreas Ostermann, Leighton Coates, Matthew P. Blakeley, Esko Oksanen, Derek T. Logan

**Affiliations:** a Biochemistry and Structural Biology, Department of Chemistry, Lund University, S-221 00 Lund, Sweden; b European Spallation Source ERIC, Box 176, S-221 00 Lund, Sweden; c Centre for Analysis and Synthesis, Department of Chemistry, Lund University, S-221 00 Lund, Sweden; d Department of Laboratory Medicine, Section MIG, Lund University, S-221 00 Lund, Sweden; e Los Alamos National Laboratory, Los Alamos, New Mexico, USA; f Jülich Centre for Neutron Science (JCNS) at Heinz Maier-Leibnitz Zentrum (MLZ), Forschungszentrum Jülich GmbH, Lichtenbergstrasse 1, 85748 Garching, Germany; g Heinz Maier-Leibnitz Zentrum (MLZ), Technische Universität München, Lichtenbergstrasse 1, 85748 Garching, Germany; h Biology and Soft Matter Division, Oak Ridge National Laboratory, Oak Ridge, Tennessee, USA; i Institut Laue–Langevin, 71 Avenue des Martyrs, 38000 Grenoble, France

**Keywords:** neutron crystallography, galectin-3C, perdeuteration, crystallogenesis

## Abstract

Galectin-3 is an important protein in molecular signalling events involving carbohydrate recognition, and an understanding of the hydrogen-bonding patterns in the carbohydrate-binding site of its C-terminal domain (galectin-3C) is important for the development of new potent inhibitors. The authors are studying these patterns using neutron crystallography. Here, the production of perdeuterated human galectin-3C and successive improvement in crystal size by the development of a crystal-growth protocol involving feeding of the crystallization drops are described. The larger crystals resulted in improved data quality and reduced data-collection times. Furthermore, protocols for complete removal of the lactose that is necessary for the production of large crystals of apo galectin-3C suitable for neutron diffraction are described. Five data sets have been collected at three different neutron sources from galectin-3C crystals of various volumes. It was possible to merge two of these to generate an almost complete neutron data set for the galectin-3C–lactose complex. These data sets provide insights into the crystal volumes and data-collection times necessary for the same system at sources with different technologies and data-collection strategies, and these insights are applicable to other systems.

## Abstract 2

Perdeuteration, purification and the growth of large crystals of the carbohydrate-recognition domain of galectin-3C are described. Five neutron diffraction data sets have been collected at four neutron sources; these are compared and two are merged.

## 1. Introduction   

Galectins are a family of proteins defined by a carbohydrate-recognition domain (CRD) of about 130 residues with affinity for galactose-containing glycans (Leffler *et al.*, 2002 ▸). Galectin-3 is one of the best known and most studied of the mammalian galectins (about 15 in total), and has multiple cellular functions in the nucleus and cytosol, inside vesicles and extracellularly. In the latter two compartments the CRD can interact with β-galactoside-containing glycoproteins and glycolipids, leading to important roles in regulation of membrane trafficking, signalling and cell adhesion (Rabinovich *et al.*, 2007 ▸; Mac­Kinnon *et al.*, 2008 ▸; Delacour *et al.*, 2009 ▸; Liu & Rabinovich, 2010 ▸; Funasaka *et al.*, 2014 ▸). For example, galectin-3 plays an important role in tumour cell adhesion, differentiation, proliferation, metastasis and angiogenesis (Liu & Rabinovich, 2005 ▸). Galectin-3 has also been found to be involved in other diseases, such as idiopathic pulmonary fibrosis, a degenerative and lethal disease that occurs primarily in middle-aged and older adults (MacKinnon *et al.*, 2012 ▸).

Galectin-3 contains a C-terminal galectin CRD (residues 113–250) and an N-terminal partially disordered peptide (residues 1–114) that may confer cross-linking upon ligand binding. Since many of the roles of galectin-3 in disease appear to require its binding to glycoconjugates mediated by the carbohydrate-binding site within the CRD, great interest has emerged in finding inhibitors of this site as potential future drugs competing with its natural ligands (Sörme *et al.*, 2002 ▸, 2005 ▸; Öberg *et al.*, 2008 ▸; Cumpstey *et al.*, 2008 ▸; Diehl *et al.*, 2010 ▸; Collins *et al.*, 2012 ▸). To this end, a large number of inhibitors have been developed, and fragments of galectin-3 containing only the CRD (galectin-3C) have been analysed using X-ray crystallography and NMR to develop even better inhibitors, also providing a unique opportunity to study the protein–ligand binding mechanism in detail from a theoretical point of view.

The carbohydrate-binding site within the CRD is solvent-exposed, hosting a significant number of water molecules when ligands are not bound, which are clearly visible in the X-ray structures, especially at 100 K (Saraboji *et al.*, 2012 ▸; Collins *et al.*, 2007 ▸). From a drug-design point of view, it is interesting to quantify the enthalpy changes that occur when ligands displace these waters, since they ultimately contribute to the total Δ*G* of binding. Furthermore, some residues, such as His158 and Glu184, play critical roles in building up a hydrogen-bond network with functional groups of the ligand. The directionality of these hydrogen bonds is extremely important to guide future inhibitor design, but unfortunately X-ray crystallography is not sensitive enough to directly observe the H atoms. Even in the X-ray crystal structure of galectin-3C in complex with lactose at 0.86 Å resolution, only about half of the H atoms were visible even at a contour level of 2σ in *m*|*F*_o_| − *D*|*F*_c_| difference maps (Saraboji *et al.*, 2012 ▸). To obtain better information on the hydrogen bonding in the carbohydrate-binding site, we have initiated neutron crystallography studies.

Here, we present methods for obtaining large crystals of perdeuterated galectin-3C in lactose-bound and glycerol-bound forms, an inhibitor-bound form and a truly apo form. Lactose is a natural ligand. Although the affinity of glycerol for galectin-3C is too weak to measure by isothermal titration calorimetry at room temperature, it has been observed to bind to galectin-3C at high concentration and to mimic part of the galactose ring (Saraboji *et al.*, 2012 ▸). Comparison of the hydrogen-bonding patterns of these two ligands and with that of water molecules in the apo structure may be instructive. We describe our experience of data collection from crystals of various volumes at four different neutron sources. Furthermore, we present the merging of data sets from two different sources into one, more complete, data set. The methods that we have used may be of value for the study of other systems.

## 2. Methods   

### 2.1. Protein expression   

Essentially fully deuterated human galectin-3C (residues 114–250, molecular mass 15.81 kDa) was heterologously expressed in *Escherichia coli* using the plasmid pGal3CRD (Lepur *et al.*, 2012 ▸). Early batches of protein were produced using glucose-d_8_ as a carbon source, but later batches used the cheaper glycerol-d_8_. A strategy of stepwise adaptation to deuterium was used. All culture media were supplemented with 50 mg l^−1^ kanamycin and 34 mg l^−1^ chloramphenicol. All cultures were grown at 37°C with shaking at 160 rev min^−1^.

#### 2.1.1. Adaptation to D_2_O   

 Cells were adapted to D_2_O using methods similar to those published previously (*e.g.* Venters *et al.*, 1995 ▸). M9 minimal medium was used for protein expression: 42.7 m*M* Na_2_HPO_4_, 22 m*M* KH_2_PO_4_, 8.6 m*M* NaCl, 2 g l^−1^ NH_4_Cl, 2 g l^−1^ glycerol, 1 m*M* MgSO_4_, 0.1 m*M* CaCl_2_, 2 g l^−1^ thiamine, 0.018 m*M* FeCl_3_. A single colony of *E. coli* BL21(DE3) cells containing the pLysS pGal3CRD plasmid grown overnight on M9 agar plates was used to inoculate 50 ml of 20% D_2_O M9 medium (with nondeuterated glycerol) to an OD_600_ of 0.1, which was then grown for 24 h. The 20% D_2_O culture was used to inoculate 50 ml 100% D_2_O M9 medium (with nondeuterated glycerol) to an OD_600_ of 0.1, and the culture was grown for 24 h.

#### 2.1.2. Adaptation to glycerol-d_8_   

The 100% D_2_O culture was used to inoculate 200 ml 100% D_2_O M9 medium with glycerol-d_8_ to an OD_600_ of 0.1. To avoid transfer of medium without glycerol-d_8_, the cells needed for inoculation were pelleted and the medium was discarded. The cell pellet was then used for inoculation and the culture was grown overnight.

#### 2.1.3. Expression of fully deuterated galectin-3C   

The 200 ml 100% D_2_O/glycerol-d_8_ culture was used to inoculate 2 × 1 l of 100% D_2_O/glycerol-d_8_ M9 medium to an OD_600_ of 0.1. At an OD_600_ of 0.5, IPTG was added to a final concentration of 0.5 m*M* and induction was continued for 12 h. Cells were harvested at 8000*g* for 20 min at 20°C. Each pellet (from 1 l culture) was resuspended in 10 ml MEPBS (10 m*M* Na_2_HPO_4_, 1.8 m*M* KH_2_PO_4_, 140 m*M* NaCl, 2.7 m*M* KCl pH 7.3, 2 m*M* EDTA, 4 m*M* β-mercaptoethanol) and stored at −80°C.

### 2.2. Preparation of soluble extract   

After thawing the frozen cell suspension on ice, one volume of MEPBS supplemented with Complete Protease Inhibitor, EDTA-free (Roche; one tablet per 30 ml final volume) was added and the cell suspension was passed twice through a French pressure cell at 124 MPa. The resulting lysate was ultracentrifuged in a 50.2 Ti rotor at 45 000 rev min^−1^ for 60 min at 4°C. The supernatant (soluble extract) was used for affinity chromatography.

### 2.3. Affinity chromatography   

An 11 ml lactosyl Sepharose column was connected to an ÄKTA avant system (GE Healthcare). The flow rate was set to 2 ml min^−1^. The column was equilibrated with 10 column volumes (CV) of MEPBS. The sample was injected and the column was washed with MEPBS (20 CV maximum). The bound protein was eluted with 5 CV MEPBS with 150 m*M* lactose. During elution, 5 ml fractions were collected. The chromatography run was performed at room temperature, while the fractions were collected at 6°C. Fractions were pooled and concentrated using an Amicon Ultra-15 3 kDa molecular-weight cutoff ultrafiltration spin column (Millipore). The buffer was exchanged for D_2_O MEPBS by diluting the concentrated sample (∼5 ml) to 15 ml with fully deuterated buffer and concentrating again, seven times in total, such that the final amount of D_2_O in the buffer was 99.9%.

**Figure 1:**
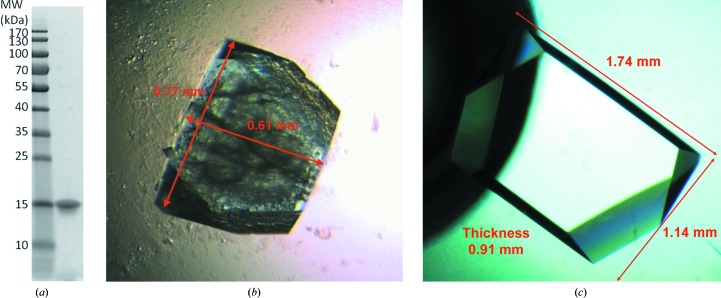
(*a*) SDS–PAGE gel of purified galectin-3C. (*b*) A typical crystal obtained by repeated macroseeding. (*c*) The 1.8 mm^3^ crystal obtained by repeated feeding of a sitting drop that was used to collect data to 1.7 Å resolution at LADI-III.

The typical yield of deuterated galectin-3C was 20 mg per litre of cell culture. The protein was filtered through a 0.22 µm filter and stored at 8°C. Its purity was estimated to be >95% by SDS–PAGE (Fig. 1 ▸ *a*) and the degree of deuteration was determined to be ≥92% by mass spectrometry, calculated as a percentage of the possible H→D substitutions.

### 2.4. Crystallization   

The crystallization conditions for galectin-3C have been established previously (Seetharaman *et al.*, 1998 ▸; Sörme *et al.*, 2005 ▸; Collins *et al.*, 2007 ▸; Diehl *et al.*, 2010 ▸; Saraboji *et al.*, 2012 ▸). The crystals belong to space group *P*2_1_2_1_2_1_. Small crystals of perdeuterated galectin-3C were obtained *via* the hanging-drop method in the following conditions: 20–28%(*w*/*v*) polyethylene glycol (PEG) 4000 or 3000, 15 m*M* β-mercaptoethanol, 0.4 *M* sodium thiocyanate, 0.1 *M* Tris–DCl pD 7.9 in D_2_O. Nondeuterated lactose was added to the protein solution to a final concentration of 10 m*M*. The final volume of the drops was 1–3 µl, and the protein:reservoir ratio was 1:1. All materials were dissolved in D_2_O. Extensive screening of different molecular-weight PEGs and their concentrations was carried out to determine the best conditions for growing well developed crystals of 0.1–0.2 mm^3^ as a starting point for seeding. The best-looking crystals (the largest, without visible cracks) were obtained at PEG 4000 or 3000 concentrations of around 24–26%.

To grow large crystals for neutron crystallography, we first tried macroseeding (Thaller *et al.*, 1981 ▸). Crystals of 0.1–0.2 mm^3^ were harvested and seeded into freshly equilibrated drops. When growth had stopped, after about a week, the crystals were harvested again and moved to new drops. The procedure was repeated a number of times (typically three times after one week and twice more at monthly intervals) until we obtained crystals of 0.4–0.5 mm^3^. Although successful, the crystals were still quite small, and the repeated macroseeding often damaged the crystals, which showed extended visible cracks over the surface. Nevertheless, the best crystals grown in this way diffracted X-rays to at least 1.0 Å resolution and were used for the first LADI-III neutron data set to 1.9 Å resolution (see below). To improve the quality, we tried other crystal-growth methods, and the most successful of these was feeding the drop with fresh protein solution. Crystals were moved to 15 + 15 µl sitting drops that had been equilibrated for one week at low PEG concentrations ranging from 12 to 15%, with one crystal per drop. The PEG concentration was sufficiently low to prevent further nucleation, but sufficiently high to allow the crystal to grow further. When the crystal had stopped growing, the sitting-drop lid was opened and 3–4 µl of additional protein plus lactose solution was added directly onto the drop. The procedure was repeated once or twice per week until a satisfactory size was obtained, in about three months. In this way we could achieve crystals of up to 1.8 mm^3^. One such crystal, measuring 1.74 × 1.14 × 0.91 mm, is shown in Fig. 1 ▸.

**Figure 2:**
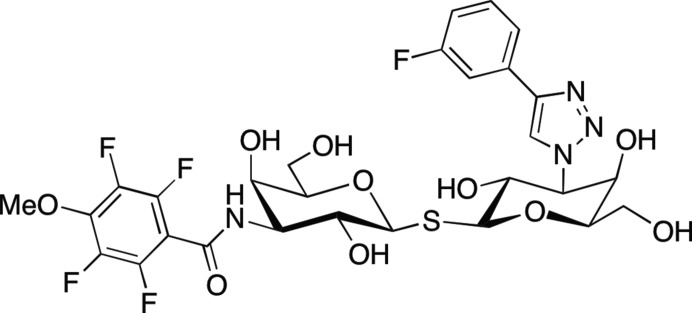
Structure of the inhibitor 3,3′-dideoxy-3′-[4-(3-fluorophenyl)-1*H*-1,2,3-triazol-1-yl]-3-(4-methoxy-2,3,5,6-tetrafluoro-benzamido)-1,1′-sulfanediyl-di-β-d-galactopyranoside.

The same procedure was adopted for growing large crystals of perdeuterated galectin-3C in complex with the thiodi­galactoside inhibitor 3,3′-dideoxy-3′-[4-(3-fluorophenyl)-1*H*-1,2,3-triazol-1-yl]-3-(4-methoxy-2,3,5,6-tetrafluoro-benzamido)-1,1′-sulfanediyl-di-β-d-galactopyranoside (Fig. 2 ▸), with the minor difference that the small crystals were moved to sitting drops to which the selected inhibitors were added to a final concentration of 5 m*M*. The feeding procedure described above was repeated, except that instead of lactose the inhibitor was added to a final concentration of 5 m*M*. Since the dissociation constants (*K*_d_) of these inhibitors are in the nanomolar range, the inhibitors naturally displaced lactose (*K*_d_ ≃ 200 µ*M*).

In order to obtain large crystals of deuterated galectin-3C with glycerol bound, a slightly different procedure was used owing to the fact that glycerol interacts only weakly with the CRD (Saraboji *et al.*, 2012 ▸). Small crystals obtained as described above were seeded into dialysis buttons initially functioning as vapour-diffusion sitting-drop supports, *i.e.* without membranes. The volume of the drop in the dialysis button was proportional to the size of the button: for 30 µl dialysis buttons, the drop size was 35 µl (25 µl reservoir + 10 µl protein). The feeding procedure was then performed identically to the lactose case, where the dialysis button was placed in an XRL plate with a reservoir of 0.5 ml. When the crystal reached a sufficient size, 10–20 µl reservoir solution was added to the dialysis button to fill it completely, and a 3.5 kDa cutoff dialysis membrane was then applied on top of the button, which was then moved to 7 ml solution as above but with 24% PEG and 10%(*v*/*v*) glycerol (1.37 *M*). The reservoir of the dialysis button was changed every week.

Crystals of apo deuterated galectin-3C were generated as follows. Firstly, large crystals of the lactose complex were grown in dialysis buttons. This was followed by a one-month dialysis against a reservoir with 10% glycerol (the same reservoir as was used to obtain glycerol-complex crystals) to displace lactose, then a one-month dialysis against the reservoir alone without glycerol.

### 2.5. Data collection   

Crystals of deuterated galectin-3C were mounted either in thin-walled quartz capillaries (Müller & Müller; from Capillary Tube Supplies Ltd, Bodmin, England) or in thick-walled quartz capillaries (Vitrocom; from CM Scientific, Silsden, England), both with internal diameter 1.5–2.0 mm, and were exposed at four different neutron sources. Firstly, several crystals of the lactose complex with a volume of about 0.3 mm^3^ (0.8 × 0.7 × 0.5 mm) were exposed for 12 h at the Protein Crystallography Station at Los Alamos National Laboratory (Kovalevsky *et al.*, 2010 ▸). The neutron wavelength range was 0.7–7 Å. These crystals showed only a few very low-resolution reflections. Similar-volume crystals from the same batch were later exposed at the LADI-III instrument of the Institut Laue–Langevin, Grenoble, France (Blakeley *et al.*, 2010 ▸) equipped with an image-plate detector (Niimura *et al.*, 1997 ▸) and showed diffraction to 1.9 Å resolution upon 16 h exposure. The wavelength range was 3.35–4.35 Å. A data set to 1.9 Å resolution was collected from 30 exposures of 16 h and 11 low-resolution exposures of 2 h in three different crystal orientations (Table 1 ▸). The crystal was rotated by 7° between exposures and the total data-collection time was 21 d. Data were indexed and integrated using *LAUEGEN* (Campbell *et al.*, 1998 ▸), wavelength-normalized using *LSCALE* (Arzt *et al.*, 1999 ▸) and scaled and merged using *SCALA* (Evans, 2006 ▸).

The 1.8 mm^3^ crystal shown in Fig. 1 ▸ allowed the collection of a data set to 1.7 Å resolution at LADI-III using exposures of 4 h. Three passes were collected in different crystal orientations. The first pass covered 90° in 19 images with an offset of 5°. The second pass covered 98° in 15 images with 7° offsets and a third pass covered 70° in seven images with 14° offsets. The neutron wavelength range used was 3–4 Å. The data were processed as described above.

A neutron time-of-flight data set using a relatively large crystal (1.0 mm^3^) of the lactose complex was collected at the MaNDi instrument at the Spallation Neutron Source, Oak Ridge National Laboratory, Tennessee, USA (Coates *et al.*, 2010 ▸, 2015 ▸). Each exposure was 10 h and used neutrons with wavelengths between 2 and 4 Å. Each exposure was separated by a φ step of 10°. At the time of data collection MaNDi was equipped with 30 of 46 possible Anger cameras. The diffraction images were processed using the *Mantid* software (Arnold *et al.*, 2014 ▸) and scaled for detector response using the Argonne National Laboratory variable-wavelength data-reduction program *ANDREV* (Schultz *et al.*, 1984 ▸). The two data sets from deuterated galectin-3C in complex with lactose, from LADI-III and MaNDi, were later merged and analysed using *XPREP* (Bruker).

Monochromatic neutron diffraction data were collected from two crystals of deuterated galectin-3C measuring around 1.0 mm^3^, one in complex with glycerol and the other in complex with the synthetic thiodigalactoside inhibitor ALN712, at the BIODIFF instrument of the FRM-II reactor in Garching, Germany, which was equipped with the same type of imaging-plate detector as at LADI-III (Niimura *et al.*, 1997 ▸). Diffraction was observed to 1.65 Å resolution for the glycerol complex and to 1.85 Å resolution for the inhibitor complex. 259 diffraction images were collected for the glycerol complex and 173 for the inhibitor complex, each with a rotation angle of 0.5° and an exposure time of 30 min. After 113 images in the case of the glycerol complex and 120 in the case of the inhibitor complex, the orientation of the crystal was changed by the use of a specially constructed goniometer head. The intensities of the reflections were integrated and scaled with a modified version of *HKL*-2000 (v.705b) adapted to neutron diffraction and *SCALEPACK* (v.2.3.8) (Otwinowski & Minor, 1997 ▸)

Crystals of apo galectin-3C have not yet been exposed to neutrons, but we have verified using X-rays (see below) that lactose can be removed from very large crystals using the two-step technique described above.

### 2.6. X-ray data collection and processing   

Room-temperature X-ray data were collected for each of the crystals from which neutron data were obtained. The lactose-2, inhibitor and glycerol complex data sets were collected at station I911-3 of the MAX-II synchrotron in Lund, Sweden (Ursby *et al.*, 2013 ▸) using a 225 mm MAR Mosaic CCD detector. For all crystals, both a low-resolution and a high-resolution data set were collected. Owing to the large size of the crystal, the low-resolution data-set exposure time was very low to avoid oversaturated spots. The high-resolution data were collected using helical data collection in order to spread out radiation damage. Data were integrated using *XDS* (Kabsch, 2010 ▸) and merged using *AIMLESS* (Evans & Murshudov, 2013 ▸) from the *CCP*4 package (Winn *et al.*, 2011 ▸). The lactose-1 X-ray data set was collected at station ID23-1 of the European Synchrotron Radiation Facility in Grenoble, France in a single pass. The lactose-3 X-ray data set was collected using a Rigaku HF-007 X-ray source and an R-AXIS IV^++^ image-plate detector at Oak Ridge National Laboratory.

### 2.7. Refinement   

All structures were refined with *phenix.refine* from the *PHENIX* package (Adams *et al.*, 2010 ▸). The starting model was the 0.86 Å resolution lactose-bound galectin-3C X-ray structure without water molecules and ligands. The structures were first refined using only X-ray data. The ligand molecules and restraints were built in *PHENIX* (Adams *et al.* 2010 ▸). Water molecules were added manually. After satisfactory *R* factors were obtained, we started joint neutron/X-ray refinement (Afonine *et al.*, 2010 ▸). The weighting between the X-ray and neutron terms was determined automatically. The optimal weighting between geometric and X-ray terms, as well as the weighting of *B*-factor restraints, was determined by multiple refinements with different weights. The neutron and electron-density maps were visualized and minor manual model rebuilding was carried out using *Coot* (Emsley *et al.*, 2010 ▸). The refined structures will be reported in a future publication.

## 3. Results and discussion   

### 3.1. Protein production   

Galectin-3 can reliably be produced in large amounts (over 100 mg per litre of cell culture) by heterologous overexpression in *E. coli*, which makes it a good system for the production of perdeuterated protein. By stepwise adaptation of *E. coli* to 100% D_2_O and the use of deuterated glucose or glycerol as carbon sources, we were able to obtain 20–50 mg of 95% pure deuterated galectin-3C deuterated to over 92% per litre of culture in a one-step affinity-purification procedure.

### 3.2. Optimization of the crystallization protocol   

**Figure 3:**
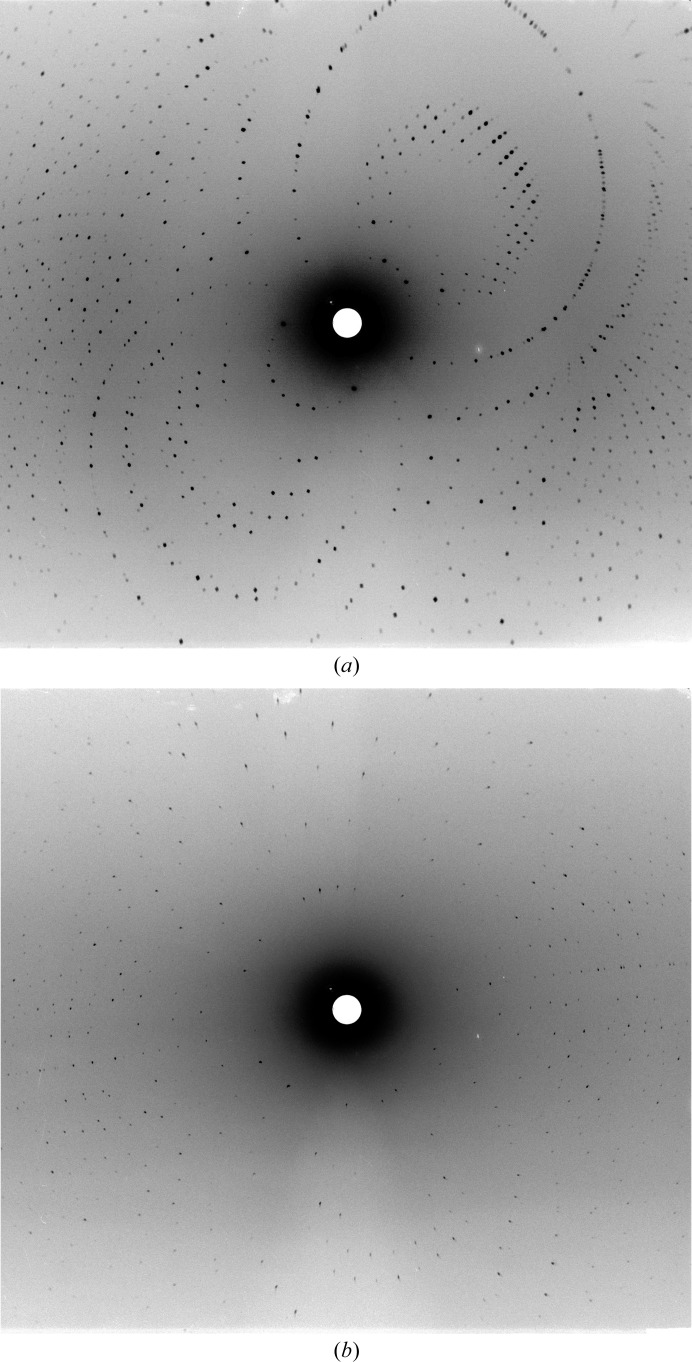
Improvement in diffraction from LADI-III. (*a*) Overall diffraction from a 4 h exposure of the crystal in Fig. 1 ▸ measuring 1.8 mm^3^. The areas around the neutron beam entrance hole in the cylindrical detector (at the left and right of the image) have been removed for clarity. The data could be integrated to 1.7 Å resolution (Table 1 ▸). (*b*) Comparable image from a crystal of 0.35 mm^3^ after 16 h exposure. Data could be integrated to 1.9 Å resolution.

Even small crystals of galectin-3C (less than 0.2 mm in the largest dimension) regularly diffract X-rays to sub-ångström resolution, but the production of crystals for neutron diffraction to high resolution required extensive optimization of the protocols. It is clear that galectin-3C crystals grown through feeding grow to a larger size and diffract significantly better compared with those grown by macroseeding. The crystals were also less cracked. The improvement is also evident if we compare the images obtained (Fig. 3 ▸) and the data quality for the two lactose complexes collected at LADI-III. A increase in crystal volume by a factor of five from 0.35 to 1.8 mm^3^ and a reduced mosaicity lowered the required exposure time by a factor of four and enabled the collection of a highly redundant data set in only 6 d. It must be noted, however, that at the time that the 1.9 Å resolution data set was collected the LADI-III instrument had only recently been relocated to its new end-position and so the process of commissioning was not yet complete. By the time that we collected the 1.7 Å resolution data set, issues regarding high background levels had been overcome through the use of additional shielding, while the flux had also improved *via* realignment of the instrument from the end of the guide to the sample position. These adjustments led to a higher signal-to-noise ratio at the sample position and will therefore also have contributed to the better data. With respect to the pure macroseeding technique, feeding has the advantage that the crystal is moved only once rather than several times, thus subjecting the crystal to less mechanical and osmotic stress. In addition, the crystal was still relatively small when moved, reducing the mechanical stress and making it less likely to crack and more likely to recover from any damage that had occurred during transfer.

Crystals of apo deuterated galectin-3C were by far the most difficult to obtain. Apo galectin-3C appears to be less soluble than when complexed with lactose or a ligand: under normal crystallization conditions a skin develops on the drops, and hypernucleation combined with precipitation is always obtained, regardless of the precipitant or reductant concentration. We tried several strategies, including different precipitants, additives and temperature screening, but the successful growth of even tiny crystals was difficult.

**Figure 4:**
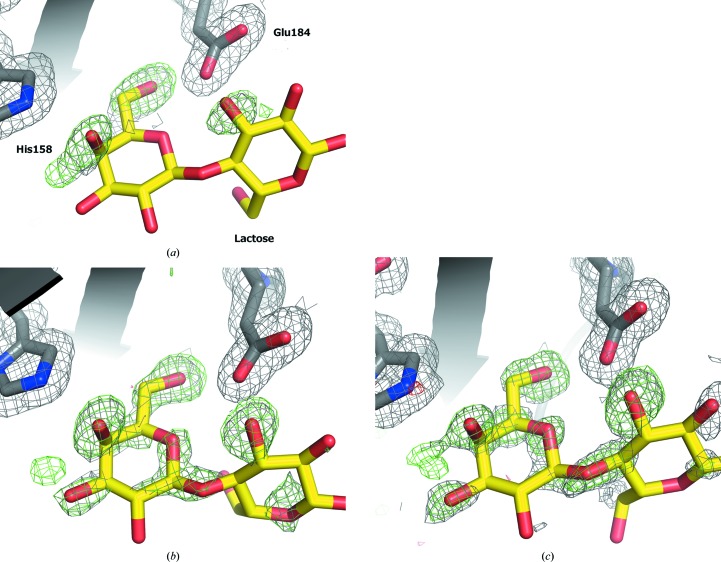
Generation of a truly apo structure by sequential dialysis against a solution containing glycerol and then only water. (*a*) Electron-density maps at 1.2 Å resolution for the apo structure after thorough dialysis, with lactose superimposed for comparison, showing only density for three water molecules. A 2*m*|*F*_o_| − *m*|*F*_c_| omit map contoured at 0.7σ is shown covering the lactose-binding site, His158 and Glu184 (grey mesh), as well as an |*F*_o_| − *m*|*F*_c_| map contoured at 3.5σ (green mesh). (*b*) Equivalent maps for the room-temperature apo X-ray structure (Saraboji *et al.*, 2012 ▸) at 1.25 Å resolution contoured at the same levels. Residual electron density for parts of lactose can still be seen. (*c*) Equivalent electron-density maps at 0.86 Å resolution for the previous apo structure at 100 K. Full analysis of the apo structure (neutron and X-ray) will be presented elsewhere.

Crystals of galectin-3C form best in the presence of lactose or synthetic ligands, which presumably reduce the global or local dynamics of the molecule in a way that is conducive to lattice formation. Obtaining a structure free of lactose is time-consuming, requiring extensive soaking or dialysis of the crystals in lactose-free solutions for up to three months, as also shown by others (Collins *et al.*, 2007 ▸). This may be owing to a highly reduced off-rate owing to the same diminished protein dynamics. The problem is presumably exacerbated by the large size of the crystals for neutron diffraction, and we found that soaking crystals for several weeks in lactose-free solution still did not remove lactose completely (Figs. 4 ▸ *a* and 4 ▸ *b*). In fact, we used a crystal treated in such a way to collect the lactose-3 data set at MaNDi. Thus, we developed a two-step method to circumvent the problem of lactose persistence, in which lactose was first replaced by glycerol and then by water. Crystals of apo galectin-3C where 20% glycerol (∼2 *M*) was used as a cryoprotectant revealed glycerol in the binding site (Saraboji *et al.*, 2012 ▸). However, contouring the electron density at lower levels suggested that there may be some residual lactose at 100 K after a few seconds of soaking (data not shown). Thus, we dialysed a large crystal against a solution containing 10% glycerol for one month, which resulted in the successful removal of all residual lactose. Although glycerol binds much more weakly than lactose, it is so abundant with respect to lactose that it successfully competes for the CRD, and the long duration of crystal growth allows it to replace lactose completely. Since some crystals are to be used to replace glycerol with water to obtain the true apo form, we grew the large crystals directly in the dialysis button to avoid the risks associated with harvesting and macroseeding.

The X-ray data that we obtained from some relatively large crystals produced in this way proved that there was no lactose or glycerol still bound to the protein, therefore providing a means of obtaining a true apo structure. An example of the electron density in the area where lactose binds is shown in Fig. 4 ▸.

### 3.3. Data quality and effects of merging   

Table 1 ▸ shows the data quality from five crystals of galectin-3C of different sizes collected at three neutron sources: two reactor-based sources and one spallation source.

Although perdeuterated, the crystals used for initial diffraction tests were, at 0.35 mm^3^, clearly too small for the source and detector at the Protein Crystallography Station (PCS), Los Alamos. We believe that the diffraction from the crystal with the limited incoming neutron flux (Kovalevsky *et al.*, 2010 ▸) was simply too weak to exceed the inherent detector noise. The perdeuteration also provides a smaller advantage at a spallation source time-of-flight instrument because the incoherent scattering background is anyway spread over many time channels. Similar-volume crystals from the same batch diffracted to 1.9 Å resolution at LADI-III and enabled the collection of a data set with good completeness and multiplicity in two weeks. This demonstrates the gain from using perdeuterated crystals at a reactor-based Laue diffractometer, where the neutron flux is high (Blakeley *et al.*, 2010 ▸) but the incoherent background is more significant.

The most dramatic improvements in data quality and collection time were achieved by using crystals of 1.8 mm^3^ produced using the feeding method. The crystal volume increased by a factor of five, which reduced the data-collection time by a factor of ∼4 (for a comparable multiplicity) but, more importantly, also improved the resolution to 1.7 Å. With stronger diffraction the exposure times can be reduced, thus reducing the losses from storage-phosphor decay in the imaging plate (Wilkinson *et al.*, 2009 ▸).

Data to even higher resolution (1.6 Å) were obtained at MaNDi in 14 d from a crystal of 1.0 mm^3^, although the completeness and multiplicity were lower than at LADI-III. This shows the advantage of the time-of-flight Laue technique, which has a lower background, for observing the weaker reflections at high resolution. The crucial difference between the two experiments at the spallation neutron sources MaNDi and PCS was the combination of the higher flux at MaNDi and a larger crystal, allowing the reflections to exceed the detector noise threshold. The lower completeness obtained could be partly explained by the fact that not all of the MaNDi detectors were available when the data were collected, thus reducing the reciprocal-space coverage.

The data collected at BIODIFF, which is a monochromatic instrument, show excellent completeness, both overall and in particular at the highest resolution, owing to the avoidance of the spatial and harmonic overlaps in the diffraction pattern that are problematic for pseudo-Laue data. The resolution for the glycerol complex rivalled that from LADI-III and MaNDi for a crystal of only 1 mm^3^. However, the relatively low flux achieved from implementation of the monochromatic method means that lower multiplicity is achievable in a given data-collection time. It is notable that the higher signal-to-background ratio at a monochromatic diffractometer allows data collections in comparable times to a reactor Laue instrument given that the crystal size is sufficient.

The data set from MaNDi was intended to be from apo deuterated galectin-3C, but problems in soaking out lactose from large crystals (which were eventually overcome) resulted in a lactose complex with a lactose occupancy of ∼0.8; therefore, we tried to combine the data sets with that obtained from LADI-III (occupancy 1.0) using *XPREP*. The internally scaled but unmerged data sets were scaled to each other and then merged. The MaNDi data set was assigned a scaling *B* factor of 58 Å^2^ relative to the LADI-III data. The resulting data set was of acceptable quality [*R*_merge_(*I*) = 20.6%], with much improved completeness (95%), particularly at high resolution (90%), and multiplicity (13). These improved statistics did not result in a dramatic improvement in the nuclear density maps, but the resulting more complete data set will result in more reliable refinement and better statistics. Furthermore, this shows proof of principle that pseudo-Laue and TOF Laue data sets from a reactor-based neutron source and a spallation source, with very different detector technologies, can be successfully combined.

### 3.4. Protein–ligand interactions   

There are several hydrogen-bonding interactions in the CRD between the protein, the ligand and the water molecules. Although the refinement and interpretation of these structures is still ongoing, some preliminary results can be described.

For the lactose-bound structure, there are three main hydroxyl groups in the lactose that can interact with protein residues in the CRD, namely O4, O6 and O3. All of these hydrogen bonds are visible in the crystals grown through feeding, while for the crystal grown though macroseeding only the hydrogen bond from O4 was clearly visible. In particular, we see that DO4 interacts with His158, while DO3 and DO4 interact with Glu184. The more complete data set obtained from merging data from ILL and MaNDi also showed that an alternative direction in which O6 can point exists, namely towards a rather disordered water molecule, with a high *B* factor compared with the surrounding residues and the rest of the structure, and is only fully visible at 0.8σ in a 2*mF*_o_ − *DF*_c_ X-ray map. The importance of this interaction is still under investigation. While the 2*m*|*F*_o_| − *D*|*F*_c_| X-ray map shows the complete lactose at 1σ, in the 2*mF*_o_ − *DF*_c_ neutron map the outer, more flexible glucose subunit of lactose is quite poorly visible and is shown in its entirety only at 0.5σ. Apart from the higher flexibility of the glucose subunit, one can also note that the lactose used in these experiments is not deuterated, which leads to cancellation effects from the H atoms that degrade the quality of the nuclear density maps.

## 4. Conclusions   

We have shown that it is possible to obtain large, high-quality perdeuterated crystals of the medically important carbo­hydrate-binding protein galectin-3C, interacting with different ligands and in a truly apo form, through macroseeding, repeated feeding and dialysis. Preliminary analysis of the structures showed a clear hydrogen-bonding network between the ligand and the protein. We showed in addition that it is possible to merge data successfully from two different neutron sources, resulting in a more complete data set. We believe that the detailed structural results, which will be presented elsewhere, may be useful for the development of better interacting ligands for galectin-3 and, in general, fruitful knowledge for the neutron crystallography and the drug-design field.

## Figures and Tables

**Table 1:** Data statistics for the galectin-3C crystals Values in parentheses are for the outer shell.

	Lactose-1	Lactose-2	Lactose-3	Merged (lactose-2 + lactose-3)	Glycerol complex	Inhibitor complex
Neutron						
Instrument	LADI-III	LADI-III	MaNDi	LADI-III/MaNDi	BIODIFF	BIODIFF
Resolution (Å)	30–1.90 (2.00–1.90)	28–1.70 (1.80–1.70)	19–1.60 (1.66–1.60)	30–1.70 (1.80–1.70)	28–1.65 (1.71–1.65)	32–1.85 (1.92–1.85)
Unit-cell parameters (Å)	*a* = 37.3, *b* = 58.5, *c* = 63.8	*a* = 37.2, *b* = 58.6, *c* = 64.1	*a* = 37.3, *b* = 58.4, *c* = 64.0	*a* = 37.2, *b* = 58.6, *c* = 64.1	*a* = 37.4, *b* = 58.5, *c* = 63.9	*a* = 37.2, *b* = 58.5, *c* = 63.8
*R*_merge_(*I*) (%)	14.7 (19.7)	16.2 (20.8)	15.5 (20.7)	20.9 (33.4)	13.5 (49.9)	20.9 (52.0)
*R*_p.i.m._(*I*) (%)	5.3 (11.1)	4.4 (7.6)	6.6 (15.0)	5.2 (13.9)	8.7 (34.3)	14.7 (40.4)
Mean *I*/σ(*I*)	11.8 (6.2)	12.4 (7.0)	12.2 (2.4)	22.2 (3.9)	5.9 (1.6)	4.1 (1.0)
Completeness (%)	86.6 (72.4)	87.1 (66.5)	81.3 (54.6)	95.8 (87.9)	94.7 (90.0)	88.7 (83.6)
No. of unique reflections	9844 (1177)	13741 (1490)	15485 (1022)	15409 (2172)	16592 (1544)	11026 (995)
Multiplicity	6.3 (3.6)	10.0 (4.9)	4.4 (1.7)	13.0 (4.9)	3.1 (2.6)	2.6 (2.2)
Time (d)	21	6	14	—	7	5
Crystal size (mm^3^)	0.35	1.8	1.0	—	1.0	1.0
X-rays						
X-ray source	ID23-1, ESRF	I911-3, MAX-II	Rigaku HF-007		I911-3, MAX-II	I911-3, MAX-II
Wavelength	0.97628	1.0000	1.5418		1.0000	1.0000
Detector	ADSC Q315R	225 mm MAR Mosaic	R-AXIS IV^++^		225 mm MAR Mosaic	225 mm MAR Mosaic
Resolution	40–1.50 (1.59–1.50)	40–1.07 (1.10–1.07)	18.7–1.67 (1.76–1.67)		40–1.15 (1.18–1.15)	40–1.25 (1.28–1.25)
Unit-cell parameters (Å)	*a* = 37.1, *b* = 58.4, *c* = 64.1	*a* = 37.0, *b* = 58.4, *c* = 63.8	*a* = 37.3, *b* = 58.4, *c* = 64.0		*a* = 37.3, *b* = 58.3, *c* = 63.9	*a* = 37.1, *b* = 58.3, *c* = 63.7
Completeness (%)	94.5 (91.1)	99.4 (96.8)	98.4 (92.9)		95.2 (92.0)	99.9 (96.2)
*R*_merge_(*I*) (%)	5.1 (55.4)	3.2 (81.7)	3.5 (14.9)		5.7 (101.8)	9.6 (98.9)
Mosaicity (°)	0.200	0.092	0.200		0.094	0.109
CC_1/2_ (%)	99.8 (78.8)	100.0 (77.2)	99.9 (96.4)		100.0 (72.6)	99.9 (56.5)
Mean *I*/σ(*I*)	15.0 (2.1)	24.7 (2.1)	14.6 (6.2)		26.2 (1.9)	25.1 (1.5)
No. of unique reflections	21776	61354	16607		47978	38974
Multiplicity	3.4	6.6	3.3		13.0	11.7
Time (min)	3	60	433		60	60
